# On the establishment of a mutant

**DOI:** 10.1007/s00285-020-01478-x

**Published:** 2020-02-26

**Authors:** Jeremy Baker, Pavel Chigansky, Peter Jagers, Fima C. Klebaner

**Affiliations:** 1 School of Mathematics, Monash University, Monash, VIC 3800 Australia; 2 Department of Statistics, The Hebrew University, 91905 Mount Scopus, Jerusalem, Israel; 3 Mathematical Sciences, Chalmers University of Technology and University of Gothenburg, 412 96 Gothenburg, Sweden

**Keywords:** Evolution models, Stochastic dynamics, Limit theorems, 92D25, 60J80, 60F17

## Abstract

How long does it take for an initially advantageous mutant to establish itself in a resident population, and what does the population composition look like then? We approach these questions in the framework of the so called Bare Bones evolution model (Klebaner et al. in J Biol Dyn 5(2):147–162, 2011. https://doi.org/10.1080/17513758.2010.506041) that provides a simplified approach to the adaptive population dynamics of binary splitting cells. As the mutant population grows, cell division becomes less probable, and it may in fact turn less likely than that of residents. Our analysis rests on the assumption of the process starting from resident populations, with sizes proportional to a large carrying capacity *K*. Actually, we assume carrying capacities to be $a_1K$ and $a_2K$ for the resident and the mutant populations, respectively, and study the dynamics for $K\rightarrow \infty$. We find conditions for the mutant to be successful in establishing itself alongside the resident. The time it takes turns out to be proportional to $\log K$. We introduce the time of establishment through the asymptotic behaviour of the stochastic nonlinear dynamics describing the evolution, and show that it is indeed $\frac{1}{\rho }\log K$, where $\rho$ is twice the probability of successful division of the mutant at its appearance. Looking at the composition of the population, at times $\frac{1}{\rho }\log K +n, n \in \mathbb {Z}_+$, we find that the densities (i.e. sizes relative to carrying capacities) of both populations follow closely the corresponding two dimensional nonlinear deterministic dynamics that starts at *a random point*. We characterise this random initial condition in terms of the scaling limit of the corresponding dynamics, and the limit of the properly scaled initial binary splitting process of the mutant. The deterministic approximation with random initial condition is in fact valid asymptotically at all times $\frac{1}{\rho }\log K +n$ with $n\in \mathbb {Z}$.

## Introduction

There has been much work in stochastic adaptive dynamics and evolutionary branching, see Dieckmann and Law (1996), Metz et al. (1996), Champagnat et al. (2002), Champagnat and Méléard (2011) and Sagitov et al. (2013), to mention just a few. Here we confine ourselves to a simple mathematical model for evolution, where an established resident population is invaded by a mutant. From that moment on, the two populations compete for resources. At the moment of invasion the resident, wild-type, population is assumed to have the size near its carrying capacity $a_1 K$. Here *K* should be thought of as large, and $a_1>0$ is fixed. The size of the mutant population is initially negligible as compared to *K*, since it starts from one individual. It has a reproductive advantage over the resident, but as its progeny grows this advantage diminishes.

We want to answer the question of how long it takes for a mutant to become established, i.e. to grow to a size comparable to the host population. And what is the population composition then? Already the simplified model of two competing populations we consider, will require new mathematical techniques and lead to insightful results. We show that the deterministic approximation with a random initial condition is valid for times $[\frac{1}{\rho }\log K] +n$ with any fixed $n\in {\mathbb {Z}}$ and a large *K*. However, unlike in the classical case on deterministic approximation (Kurtz 1970; Barbour 1980), some stochasticity remains and enters as a random initial condition.

### The Bare Bones evolutionary model

This simple but basic model of species reproducing under interaction with their environment was introduced in Klebaner et al. (2011). It builds upon asexual binary splitting and evolves in discrete time. Thus, each individual either gets two children in the next generation or none. However, interaction with environment and population size is allowed—in contrast to classical stochastic approaches—but drastically condensed. Following the idea of Malthus, populations reach sizes proportional to available resources, and we assume that the the habitat is characterised by a *carrying capacity*, $K>0$, thought of as large. Given the population size, individuals reproduce independently. Initially only the resident, wild-type, population is present and, at population size *z*, the individual probability of successful splitting is taken to be $a_1 K/(a_1 K + z)$. Here $a_1$ is a constant, which determines the population size at its macroscopic (quasi-)equilibrium: when $z=a_1 K$, the probability of splitting is 1/2. On the average, thus, a population of this size produces one child per individual. As a result, the population size fluctuates around this (quasi-)steady state for what is presumably a very long time, cf. Jagers and Klebaner (2011).

In that stage, the population will experience its first mutation giving rise to a new population. The new, mutant population starts from a single individual, its ancestor. The basis of adaptive dynamics can then be said to be furnished by the branching mechanism, which forces the new population to either die out or else embark on exponential growth, in which case the old resident dies out, or the two populations will coexist for a time span that turns out to be exponential in the carrying capacity.

Mathematically, this dynamics can be described as follows. The branching process starts from a pair of positive integers ${\mathbf {Z}}(0)=\big (Z_1(0),Z_2(0)\big )$, the first component denoting the size of the resident and the second that of the mutant population, at time 0, when the mutation appears. We assume that the established original population is at equilibrium at the moment of invasion $n=0$, $Z_1(0)=[a_1K]$, and $Z_2(0)=1$. Each population develops by binary splitting with probabilities dependent on the numbers of cells, with transitions from generation *n* to $n+1$ described by the recursion

1 $$\begin{aligned} {{\mathbf {Z}}}(n+1)=\Big ( Z_1(n+1),Z_2(n+1)\Big ) =\left( \sum _{k=1}^{Z_{1}(n)}\xi _1 (n,k), \sum _{k=1}^{Z_{2}(n)}\xi _2 (n,k)\right) . \end{aligned}$$

The random variables $\xi _i(n,k)\in \{0,2\}$ are independent, given the preceding, and only depend upon the last generation ${\mathbf {Z}}(n)$, with probabilities

2 $$\begin{aligned} \begin{aligned} {\mathbb {P}}\Big ( \xi _1(n,k)=2| {\mathbf {Z}}(n)\Big )= & {} \frac{a_{1}K}{a_{1}K+Z_1(n)+\gamma Z_2(n)},\\ {\mathbb {P}}\Big ( \xi _2(n,k)=2| {\mathbf {Z}}(n)\Big )= & {} \frac{a_{2}K}{a_{2}K+\gamma Z_1(n)+Z_2(n)}, \end{aligned} \end{aligned}$$

where $a_2>0$ is the parameter, which controls the mutant equilibrium population size, and $\gamma$ is the interaction coefficient, assumed to satisfy $0<\gamma <1$. The biological meaning of $\gamma$ is that cells of one type encroach less upon the reproduction of the other cell type than do cells of the same type. That $\gamma$ is the same in both probabilities means that influence is symmetric between the cell types.

In the absence of mutants, the established population thus has a critical reproduction, whereas the mutant population starts supercritically, provided $a_2>\gamma a_1$, as is assumed throughout the paper, see (C) below.

### Stochastic nonlinear dynamics for the evolution of the density

Important insights into the behaviour of populations with state dependent reproduction and large carrying capacity is provided by their *density process* (Klebaner 1984, 1993). It allows representation of the process as having stochastic nonlinear dynamics, which can be separated into a deterministic part and a random perturbation. This is useful not only for the mathematical analysis but also for the biological interpretation.

The density process is the population sizes relative to *K*

$$\begin{aligned} {\mathbf {X}}(n)=\big (X_1(n),X_2(n)\big ):=\big (Z_1(n)/K,Z_2(n)/K\big ). \end{aligned}$$

Note that the splitting probabilities (and hence the offspring distributions) in (2) are in fact functions of the density; denoting the density state by ${\mathbf {x}}=(x_1,x_2)$ we see that

$$\begin{aligned} {\mathbb {P}}\left( \xi _1(n,k)=2| {\mathbf {X}}(n) = {\mathbf {x}}\right)&= \frac{a_{1}}{a_{1}+x_{1}+\gamma x_{2}}, \\ {\mathbb {P}}\left( \xi _2(n,k)=2| {\mathbf {X}}(n) = {\mathbf {x}}\right)&=\frac{a_{2}}{a_{2}+\gamma x_{1}+x_{2}}. \end{aligned}$$

Accordingly, the offspring mean ${\mathbf {m}}({\mathbf {x}})= \big (m_1( {{\mathbf {x}}}), m_2( {{\mathbf {x}}})\big )$ at ${\mathbf {x}}$ is also a function of the density

$$\begin{aligned} m_{1}({\mathbf {x}})&={\mathbb {E}}\big (\xi _1(n,k)| {\mathbf {X}}(n)= {{\mathbf {x}}}\big )=\frac{2a_{1}}{a_{1}+x_{1}+\gamma x_{2}}, \\ m_{2}({\mathbf {x}})&={\mathbb {E}}\big (\xi _2(n,k)| {\mathbf {X}}(n)= {{\mathbf {x}}}\big )=\frac{2a_{2}}{a_{2}+\gamma x_{1}+x_{2}}. \end{aligned}$$

The underlying deterministic dynamics

3 $$\begin{aligned} {\mathbf {x}}(n+1)= {\mathbf {f}}\big ( {\mathbf {x}}(n)\big ), \end{aligned}$$

is determined by the function ${\mathbf {f}}({\mathbf {x}})=\big (f_1({\mathbf {x}}), f_2({\mathbf {x}})\big )$,

4 $$\begin{aligned} \begin{aligned} f_1( {\mathbf {x}})&= x_1 m_1({\mathbf {x}})= \frac{2x_1a_1}{a_1+x_1+\gamma x_2}, \\ f_2({\mathbf {x}})&= x_2 m_2({\mathbf {x}}) = \frac{2x_2a_2}{a_2+\gamma x_1+ x_2}. \end{aligned} \end{aligned}$$

This can be easily seen from (1) by writing the density process as

5 $$\begin{aligned} \begin{aligned} X_1(n+1)&= X_1(n)m_{1}\big ( {\mathbf {X}}(n)\big ) + \frac{1}{K}\sum _{j=1}^{KX_1(n)}\Big (\xi _1(n,j)-m_{1}\big ( {\mathbf {X}}(n)\big )\Big ) \\ X_2(n+1)&= X_2(n) m_2\big ({\mathbf {X}}(n)\big ) + \frac{1}{K}\sum _{j=1}^{KX_2(n)}\Big (\xi _2(n,j) -m_{2}\big ( {\mathbf {X}}(n) \big )\Big ). \end{aligned} \end{aligned}$$

The first term on the r.h.s. of (5) gives the deterministic dynamics (3), and the second term acts as the random perturbation,

6 $$\begin{aligned} {\mathbf {X}}(n+1) ={\mathbf {f}}\big ({\mathbf {X}}(n)\big )+\frac{1}{\sqrt{K}} \varvec{\eta } (n+1,{\mathbf {X}}_n), \end{aligned}$$

with

$$\begin{aligned} \eta _i(n+1, {\mathbf {x}})=\frac{1}{\sqrt{K}}\sum _{j=1}^{K x_i}\big (\xi _i(n,j)-m_i({\mathbf {x}})\big ),\quad i=1,2. \end{aligned}$$

These random variables have zero mean and variance $4x_ip_i({\mathbf {x}})\big (1-p_i({\mathbf {x}})\big )$, where $p_i({\mathbf {x}})$ are the splitting probabilities. Therefore the random noise term in (6) is of order $1/\sqrt{K}$ and the density process can indeed be viewed as generated by a nonlinear dynamical system, perturbed by a small random disturbance.

Note that in the view of the above discussion, the trajectory of the deterministic system (3) depends on *K* through the initial condition ${\mathbf {x}}^K(0)=\big (\frac{[a_1K]}{K}, \frac{1}{K}\big )$. Similarly, the process generated by the stochastic dynamics (6), depends on *K* through ${\mathbf {X}}^K(0)=\big (\frac{[a_1K]}{K}, \frac{1}{K}\big )$ and the noise term. Whenever appropriate, we will leave this dependence implicit, omitting it from the notation.

### Deterministic dynamics

If we neglect the small random noise in (6), we obtain the deterministic dynamics (3). Fixed points (solutions to ${\mathbf {f}}({\mathbf {x}})= {\mathbf {x}}$) play an important role in the behaviour of such systems. The trajectories are repelled from the unstable fixed points and attracted to the stable ones. Our system, generated by the function ${\mathbf {f}}(\cdot )$ in (4), has four fixed points,

7 $$\begin{aligned} \begin{array}{llll} &{}{\mathbf {x}}^{\mathrm {ex}}=(0,0) &{} &{}\text {(total extinction equilibrium)}\\ &{}{\mathbf {x}}^{\mathrm {re}}=(a_1,0) &{} &{} \text {(resident equilibrium in absence of mutant)} \\ &{}{\mathbf {x}}^{\mathrm {mu}} = (0,a_2) &{} &{} \text {(mutant equilibrium in absence of resident)} \\ &{}{\mathbf {x}}^{\mathrm {co}} =\Big (\frac{a_1-\gamma a_2}{1-\gamma ^2}, \frac{a_2-\gamma a_1}{1-\gamma ^2}\Big ) &{} &{} \text {(coexistence equilibrium)} \end{array} \end{aligned}$$

Since we are concerned with both populations, the relevant case is when both coordinates of ${\mathbf {x}}^{\mathrm {co}}$ are nonnegative. This is true if the following *co-existence* condition holds

C $$\begin{aligned} a_1-\gamma a_2>0,\;\;a_2-\gamma a_1>0. \end{aligned}$$

It is easy to see by examining the Jacobian matrix $\nabla {\mathbf {f}}({\mathbf {x}})$, see (22) below, that the point ${\mathbf {x}}^{\mathrm {co}}$ is stable, and ${\mathbf {x}}^{\mathrm {ex}}$ unstable. The points ${\mathbf {x}}^{\mathrm {re}}$ and ${\mathbf {x}}^{\mathrm {mu}}$ are saddle points, that is, stable in one direction and unstable in another. In our theory the point ${\mathbf {x}}^{\mathrm {re}}=(a_1,0)$ plays a special role due to proximity of the initial condition $\big (\frac{[a_1 K]}{K},\frac{1}{K}\big )$. In the absence of a mutant, $a_1$ is the stable equilibrium for the resident population, and 0 is unstable for the mutant population.

### The large capacity limit of the stochastic dynamics

A rigorous treatment for neglecting small noise is given by the classical results in perturbation theory of dynamical systems, see e.g. Kurtz (1970), Barbour (1980), Freidlin and Wentzell (1984) and Kifer (1988). They assert that as the noise converges to zero, that is, when $K\rightarrow \infty$, the trajectory ${\mathbf {X}}^K(n)$ of the stochastic system (6) converges on any *bounded* time interval to that of the deterministic dynamics (3), started from the initial condition ${\mathbf {x}}(0) = \lim _{K\rightarrow \infty }{\mathbf {X}}^K(0)$. Namely, for an arbitrary but fixed integer *N*,

8 $$\begin{aligned} \max _{n \le N} \big |{\mathbf {X}}^K(n)-{\mathbf {x}}(n)\big |\xrightarrow [K\rightarrow \infty ]{{\mathbb {P}}}0. \end{aligned}$$

In our setup, the initial condition turns out to be the fixed point ${\mathbf {x}}^{\mathrm {re}}$,

$$\begin{aligned} {\mathbf {x}}(0) =\lim _{K\rightarrow \infty } {\mathbf {X}}^K(0)=\lim _{K\rightarrow \infty } \big (\tfrac{[a_1 K]}{K},\tfrac{1}{K}\big )= (a_1,0)={\mathbf {x}}^{\mathrm {re}}. \end{aligned}$$

Therefore the corresponding limit trajectory is constant, ${\mathbf {x}}(n)={\mathbf {x}}^{\mathrm {re}}$ for all $n=1,2,\dots$ Consequently, the limit (8) fails to provide any information on the transition to a new coexistence equilibrium. We shall see that if such a transition occurs, it becomes visible much later, at a time increasing with *K*, in fact, of order $\log K$.

Recently, limit theorems, capable of capturing this transition, were obtained in Barbour et al. (2015, 2016), Chigansky et al. (2018) and Baker et al. (2018). They involve a time shift which grows logarithmically in *K*. In Barbour et al. (2015) this shift is *random* and the process ${\mathbf {X}}^K(n)$ is approximated by the trajectory of the deterministic system (3) with a random shift. We have learnt from a referee that a precursor to random shift theory in Barbour et al. (2015) in the context of epidemic models can be found in Metz (1978), where precise conjectures were stated and later proved in an unpublished manuscript for the simple SIR epidemic model, (Altmann 1993; Mollison 1995).

In Barbour et al. (2016), Chigansky et al. (2018) and Baker et al. (2018), the shift is *deterministic*, and ${\mathbf {X}}^K(n)$ converges to a trajectory of (3), started from a *random* initial condition. While the two approaches, the random shift and the random initial condition, are related, they are not equivalent. The main building block in the random initial condition theory is a certain scaling limit of the deterministic flow, which does not appear in the random shift theory. Existence of this limit was so far established only in the one dimensional case.

This work is the first such result in two dimensions. Having established it, we can complement the “random shift” picture in Barbour et al. (2015) with that of “random initial condition” for the Bare Bones model. Recently heuristics for similar random initial conditions for selective sweeps in large populations in one dimension were given in Martin and Lambert (2015). Other stochastic approaches involving carrying capacity can be found in Lambert (2005, 2006).

## Main results

In what follows we consider the stochastic process ${\mathbf {X}}^K(n)$ generated by (6) or, equivalently, by (1). As mentioned in Introduction, the resident population initially has a critical reproduction, and is at equilibrium, when a single mutant appears, so that ${\mathbf {X}}^K(0)=\big (\frac{[a_1 K]}{K},\frac{1}{K}\big )\approx {\mathbf {x}}^{\mathrm {re}}=(a_1,0)$. Even though the probability of a mutant present at any time *n* is positive, ${\mathbb {P}}(X_2^K(n)>0)>0$, we do not say that it established itself until its numbers are proportional to its carrying capacity, in other words proportional to *K*. This can be formalized as

$$\begin{aligned} \liminf _{K\rightarrow \infty } X_2^K(n) >0. \end{aligned}$$

For example, as we have seen above ${\mathbf {X}}^K(n)\rightarrow {\mathbf {x}}^{\mathrm {re}}=(a_1,0)$ for any fixed *n* as $K\rightarrow \infty$. This conveys that the mutant is not established by any fixed time *n*. We show however, that it may establish itself at a time, which grows logarithmically with *K*. More precisely, we prove that at time $[b\log K]$ with a certain constant *b*,

9 $$\begin{aligned} \liminf _{K\rightarrow \infty } X_2^K\big ([b\log K]\big ) >0,\;\;\left( \text{ and }\,\, \limsup _{K\rightarrow \infty } X_2^K\big ([b\log K]\big )<\infty \right) , \end{aligned}$$

whereas for $$0< r < b$$,

10 $$\begin{aligned} \lim _{K\rightarrow \infty }{\mathbf {X}}^K([r\log K])\rightarrow {\mathbf {x}}^{\mathrm {re}}, \end{aligned}$$

and, therefore, $X_2^K \big ([r\log K]\big ) \xrightarrow [K\rightarrow \infty ]{{\mathbb {P}}} 0,$ in particular.

The logarithmic order of time of the mutant’s establishment can be roughly explained as follows. As the process starts near ${\mathbf {x}}^{\mathrm {re}}=(a_1,0)$, the state dependent splitting probabilities can be approximated, at least initially, by their values at ${\mathbf {x}}^{\mathrm {re}}$, giving probabilities of division 1/2 and $a_2 /( a_2+\gamma a_1)$ for the resident and the mutant populations respectively. Note that due to coexistence condition (C), the mutant process is supercritical with mean

$$\begin{aligned} \rho = \frac{2a_2}{a_2+\gamma a_1} >1. \end{aligned}$$

Hence it grows at the rate $\rho ^n$, and it takes time

$$\begin{aligned} b\log K+O(1), \text { with } b:=\frac{1}{\log \rho } \end{aligned}$$

for it to grow to the size proportional to *K*, as $K\rightarrow \infty$. In fact, this heuristics is correct, and made precise in the following result, which implies both (9) and (10). We denote the fractional part of $x\in {\mathbb {R}}_+$ by $\{x\}$.

### Theorem 1

There exist a non-degenerate scalar random variable $W\ge 0$ and a function ${\mathbf {H}}({\mathbf {x}})$, whose entries are positive on the open half-plane ${\mathbb {R}}\times {\mathbb {R}}_+$, such that

11 $$\begin{aligned} {\mathbf {X}}^K \big ([b\log K]\big ) - {\mathbf {H}}\left( \rho ^{-\{\log _\rho K\}}\begin{pmatrix} 0 \\ W \end{pmatrix} \right) \xrightarrow [K\rightarrow \infty ]{{\mathbb {P}}} \mathbf{0}. \end{aligned}$$

In particular, along the subsequence of exact powers $K_j = \rho ^j$, $j\in {\mathbb {N}}$,

$$\begin{aligned} {\mathbf {X}}^{K_j} \big (b\log K_j\big ) \xrightarrow [j\rightarrow \infty ]{{\mathbb {P}}} {\mathbf {H}}\left( \begin{pmatrix} 0 \\ W \end{pmatrix} \right) . \end{aligned}$$

Let us now detail about the random variable *W* and the function ${\mathbf {H}}(\cdot )$ appearing in this theorem. The approximate mutant process, mentioned in the heuristic explanation above, has the same splitting probability as the mutant component of ${\mathbf {Z}}(n)$ at ${\mathbf {x}}^{\mathrm {re}}$. More precisely, it is a supercritical Galton-Watson binary splitting, started with a single ancestor, $Y(0)=Z_2(0)=1$, and for $n\ge 1$ defined iteratively by

12 $$\begin{aligned} Y(n+1) = \sum _{j=1}^{Y(n)} \zeta (n,j), \end{aligned}$$

where the offsprings $\zeta (n,j)\in \{0,2\}$ are i.i.d. random variables with the constant splitting probability ${\mathbb {P}}\big (\zeta (n,j) = 2\big )=\rho /2$.

It is well known that $W(n) = \rho ^{-n} Y(n)$ is a non-negative martingale. As such it converges almost surely to a limit,

$$\begin{aligned} W=\lim _{n\rightarrow \infty } W(n), \end{aligned}$$

which is the random variable appearing in (11).

The function ${\mathbf {H}}(\cdot )$ in Theorem 1 is the limit of the *n*-fold iterated map ${\mathbf {f}}^n(\cdot )$ along the unstable manifold of the dynamics in (3).

### Theorem 2

Under the basic assumptions stated, the limit

13 $$\begin{aligned} {\mathbf {H}}({\mathbf {x}})= \lim _{n\rightarrow \infty } {\mathbf {f}}^n({\mathbf {x}}^{\mathrm {re}}+{\mathbf {x}}/\rho ^n), \quad {\mathbf {x}}\in {\mathbb {R}}\times {\mathbb {R}}_+ \end{aligned}$$

exists, and the convergence is uniform on compacts.

**Fig. 1:**
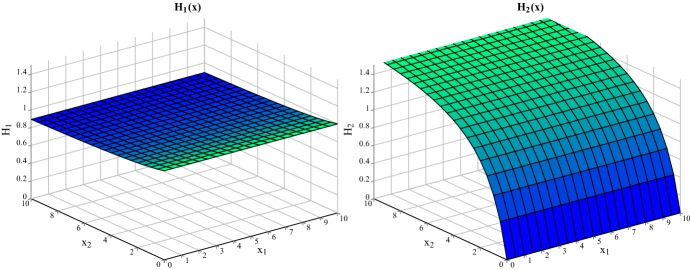
Numerical approximation of the function ${\mathbf {H}}({\mathbf {x}})$

### Remark 1

(1) It is easy to see that ${\mathbf {H}}(\cdot )$ solves the Abel functional equation ${\mathbf {H}}({\mathbf {x}})={\mathbf {f}}({\mathbf {H}}({\mathbf {x}}/\rho ))$, subject to ${\mathbf {H}}(\mathbf 0)=x^{\mathrm {re}}$. While much is known of such equations in one dimension, in higher dimensions the theory is more involved.

(2) Numerical calculations indicate that ${\mathbf {H}}({\mathbf {x}})$ is constant with respect to the resident population component $x_1$, see Fig. 1. This is consistent with the criticality of that population at the density $a_1$: the global stability of the monomorphic dynamics makes those perturbations shrink to 0 when ${\mathbf {f}}$ is iterated.

**Fig. 2:**
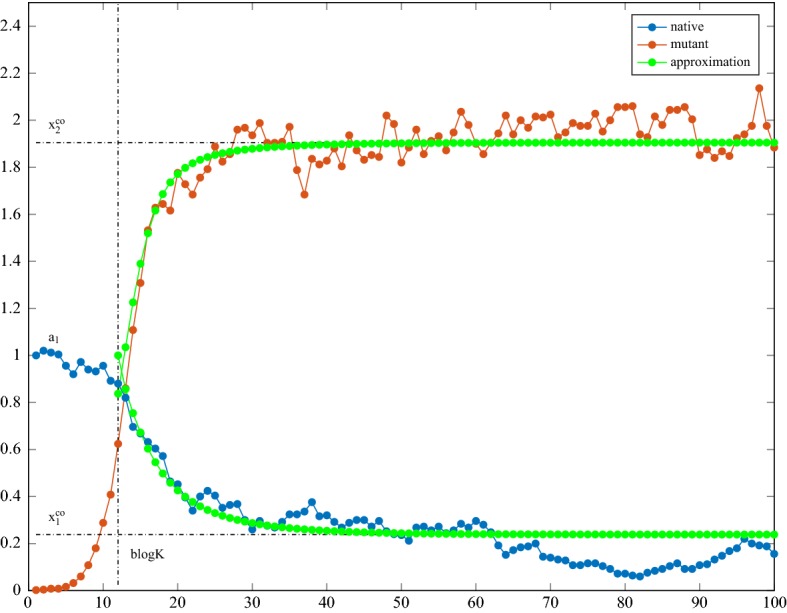
The density process versus the approximation with the random initial condition

The next result describes the density process after establishment of the mutant, at times $[b\log K]+n$, $n= 1, 2, \dots$; it shows that the population composition is governed by the deterministic nonlinear dynamics ${\mathbf {f}}^n$ started at the random point, as illustrated on Fig. 2. Furthermore, it equally holds when *n* is a negative integer. Denote the random vector appearing in Theorem 1 by

$$\begin{aligned} \varvec{\chi }(K) ={\mathbf {H}}\left( \rho ^{-\{\log _\rho K\}}\begin{pmatrix} 0 \\ W \end{pmatrix} \right) . \end{aligned}$$

### Corollary 1

For any $$n\in {\mathbb {Z}}$$,

$$\begin{aligned} {\mathbf {X}}^K([b\log K]+n)- {\mathbf {f}}^n (\varvec{\chi }(K) ) \xrightarrow [K\rightarrow \infty ]{{\mathbb {P}}}0. \end{aligned}$$

The next corollary to Theorem 1 answers the question what is the probability of the successful establishment of the mutant? Since the argument is short we present it here. It is known that ${\mathbb {P}}(W=0)$ is exactly the extinction probability of the Galton-Watson process *Y*(*n*). It is easily calculated to be $2/\rho -1$. But on the event $\{W=0\}$, ${\mathbf {H}}\big ((0,W)\big )={\mathbf {H}}((0,0))={\mathbf {x}}^{\mathrm {re}}$. Since on the complimentary event $W>0$, and ${\mathbf {H}}_2((0,w)) >0$ for $w>0$, we have the following corollary of (11).

### Corollary 2

With probability $2(1-\rho ^{-1})$ the mutant establishes itself alongside the large original population.

## Proofs

### A preview

The proof is inspired by the observation that supercritical populations, which start from a small number of individuals and develop on a habitat with a large but bounded capacity, grow initially as the Galton-Watson supercritical branching and then follow closely a deterministic curve, determined by the underlying nonlinear dynamics. This heuristics dates back at least to 50’s, e.g., Kendall (1956) and Whittle (1955), and the already mentioned (Metz 1978). A rigorous proof for epidemics is given in Mollison (1995), and in a wider context the rigorous implementations are relatively recent, see Barbour et al. (2015, 2016) and Chigansky et al. (2018).

Let us briefly sketch the ideas. The main ingredient is the Galton-Watson branching process ${\mathbf {Y}}(n)$, whose components mimic the behaviour of those of ${\mathbf {Z}}(n)$ at the moment of mutant’s appearance, that is, around the equilibrium point ${\mathbf {x}}^{\mathrm {re}}$. Thus its first, resident component $Y_1(n)$ is critical and starts at $[a_1K]$ and its second, mutant component $Y_2(n)$ is supercritical with the offspring mean $\rho$, and it starts from a single individual. The two processes ${\mathbf {Z}}(n)$ and ${\mathbf {Y}}(n)$ are constructed on the same probability space and are coupled in such a way, that they remain close at least until time $n_c = [\log _\rho K^c]$ with some fixed constant $c\in (1/2,1)$.

Following the above heuristics the density ${\mathbf {X}}(n) = {\mathbf {Z}}(n)/K =: \overline{{\mathbf {Z}}}(n)$ is approximated by gluing the linearized stochastic process with the deterministic nonlinear dynamics,

$$\begin{aligned} {\widetilde{{\mathbf {Z}}}}(n) = {\left\{ \begin{array}{ll} \overline{{\mathbf {Y}}}(n) &{} n\le n_c \\ {\mathbf {f}}^{n-n_c}\big (\overline{{\mathbf {Y}}}(n_c)\big ) &{} n>n_c \end{array}\right. } \end{aligned}$$

where $\overline{{\mathbf {Y}}}(n) := {\mathbf {Y}}(n)/K$ is the density of the Galton-Watson branching. The assertion (11) of Theorem 1 follows if we show that

1. the process ${\widetilde{{\mathbf {Z}}}}(n)$ does indeed approximate the target density $\overline{{\mathbf {Z}}}(n)$ at time $n=[\log _\rho K]=\big [\frac{1}{\log \rho } \log K\big ]=n_1$,
  14 $$\begin{aligned} \overline{{\mathbf {Z}}}(n_1) - {\widetilde{{\mathbf {Z}}}}(n_1) \xrightarrow [K\rightarrow \infty ]{{\mathbb {P}}} 0, \end{aligned}$$
2. and the approximation ${\widetilde{{\mathbf {Z}}}}(n)$ behaves asymptotically as claimed in Theorem 1,
  15 $$\begin{aligned} {\widetilde{{\mathbf {Z}}}}(n_1) - {\mathbf {H}}\left( \rho ^{-\{\log _\rho K\}}\begin{pmatrix} 0 \\ W \end{pmatrix} \right) \xrightarrow [K\rightarrow \infty ]{{\mathbb {P}}}0. \end{aligned}$$

The main technical difficulty in proving (14) is to control the difference $\overline{{\mathbf {Z}}}(n) - {\widetilde{{\mathbf {Z}}}}(n)$ on the time interval $[0,n_1]$, which itself grows with *K*. It turns out that the usual technique, based on straightforward linearization of the dynamics, does not provide bounds sharp enough in this case. Instead we construct a suitable coupling in Sect. 3.3, which involves several additional auxiliary Galton-Watson processes.

The key to the limiting expression in (15) is the representation

16 $$\begin{aligned} \begin{aligned}&{\mathbf {f}}^{n_1-n_c} \big (\overline{{\mathbf {Y}}}(n_c)\big ) \\&\quad ={\mathbf {f}}^{n_1-n_c} \Big ({\mathbf {x}}^{\mathrm {re}}+ \rho ^{-\{\log _\rho K\}}\rho ^{-(n_1-n_c)}\rho ^{-n_c}\big ( {\mathbf {Y}}(n_c)-K {\mathbf {x}}^{\mathrm {re}} \big )\Big ). \end{aligned} \end{aligned}$$

It shows that the convergence in Theorem 1 follows once we prove the limit of Theorem 2 and check that

17 $$\begin{aligned} \rho ^{-n_c}\big ( {\mathbf {Y}}(n_c)-K {\mathbf {x}}^{\mathrm {re}} \big )\xrightarrow [K\rightarrow \infty ]{{\mathbb {P}}}(0,W). \end{aligned}$$

The random variable *W* is the martingale limit of the supercritical branching $Y_2(n)$, cf. (12). The most challenging element of the proof of this part is convergence (13), see Sect. 3.2 below. Previously, it has been proved in Chigansky et al. (2018) in dimension one, and analysis in higher dimensions, in our case two, requires a completely different approach. When convergence (11) is proved, the assertion of Corollary 1 follows by continuity of ${\mathbf {f}}(\cdot )$.

The limit in equation (10) can be proved in a similar way: note that in this case, cf. (16)

$$\begin{aligned}&{\mathbf {f}}^{n_r-n_c} \big (\overline{{\mathbf {Y}}}(n_c)\big ) \\&\quad = {\mathbf {f}}^{n_r-n_c} \Big ({\mathbf {x}}^{\mathrm {re}}+ \rho ^{-\{\log _\rho K\}}\rho ^{-(n_r-n_c)}\rho ^{-(n_1-n_r)}\rho ^{-n_c}\big ( {\mathbf {Y}}(n_c)-K {\mathbf {x}}^{\mathrm {re}} \big )\Big ), \end{aligned}$$

where, in view of (17),

$$\begin{aligned} \rho ^{-(n_1-n_r)}\rho ^{-n_c}\big ( {\mathbf {Y}}(n_c)-K {\mathbf {x}}^{\mathrm {re}} \big )\xrightarrow [K\rightarrow \infty ]{{\mathbb {P}}} 0. \end{aligned}$$

We omit the proof of this part, which closely follows that of Theorem 1 with obvious adjustments.

### The limit $\mathbf {H(x)}$

In this section we construct limit (13), by means of a convergent telescopic series.

#### An auxiliary recursion in dimension one

Let us start with an auxiliary one dimensional quadratic recursion

18 $$\begin{aligned} x_{m,n} = \rho x_{m-1,n}\big (1+C x_{m-1,n}\big ), \quad m = 1,\dots ,n \end{aligned}$$

subject to initial condition $x_{0,n}=x/\rho ^n$ with $x>0$, where $C\ge 0$ and $\rho >1$ are constant coefficients. In what follows we will need the following estimate on its solution.

##### Lemma 1

There exists a finite function $\psi :{\mathbb {R}}_+\mapsto {\mathbb {R}}_+$, such that

19 $$\begin{aligned} x_{m,n} \le \psi (x) \rho ^{m-n}, \quad m=1,\dots ,n. \end{aligned}$$

##### Proof

Let us first note that no generality will be lost if $C=1$ is assumed. Indeed, if (18) is multiplied by *C*, the recursion

$$\begin{aligned} {\widetilde{x}}_{m,n} = p({\widetilde{x}}_{m-1,n}), \quad m=1,\dots ,n \end{aligned}$$

with $p(x)= \rho x(1+x)$ is obtained for the rescaled sequence ${\widetilde{x}}_{m,n} = C x_{m,n}$. Hence if (19) holds for ${\widetilde{x}}_{m,n}$ with some ${\widetilde{\psi }}(x)$, then it holds for $x_{m,n}$ with $\psi (x) := C^{-1} {\widetilde{\psi }}(Cx)$. From here on we set $C=1$.

Since $x_{m,n} \ge \rho x_{m-1,n},$ proving the desired bound amounts to showing

20 $$\begin{aligned} \sup _n x_{n,n}<\infty , \quad \forall x\ge 0. \end{aligned}$$

To this end, consider the Schröder functional equation

21 $$\begin{aligned} \phi (f(x))=s\phi (x), \quad x\in [0,\infty ) \end{aligned}$$

where $s=:1/\rho \in (0,1)$ and $f(x)=\sqrt{\frac{1}{4} + sx}-\frac{1}{2}$ is the inverse of the parabola $p(\cdot )$ on ${\mathbb {R}}_+$. The function *f*(*x*) satisfies the following conditions

1. *f* is continuous and strictly increasing on $[0,\infty )$
2. $$f(0)=0$$ and $$0<f(x)<x$$ for $$0<x<\infty $$
3. $$f(x)/x\rightarrow s$$ as $$x\rightarrow 0+$$
4. *f*(*x*) is concave (and therefore *f*(*x*)/*x* is decreasing on ${\mathbb {R}}_+$)
5. for all $$\delta >0$$
  $$\begin{aligned} \int _0^\delta \frac{|f(x)-sx|}{x^2}dx<\infty . \end{aligned}$$

Under these conditions (Seneta 1969) shows that the limit

$$\begin{aligned} \phi (x):= \lim _n \frac{f^n(x)}{s^n}, \quad x\in [0,\infty ) \end{aligned}$$

exists, solves (21) and satisfies the following properties

- $0<\phi (x)<\infty$ on $(0,\infty )$ (nontrivial limit), $\phi (0+)=0$
- $\phi (x)/x$ is monotone on $(0,\infty )$
- $$ \phi '(0+)=1 $$
- $$\phi (x)$$ is invertible^1^

Changing the variable in (21) to $y=f(x)$, we get

$$\begin{aligned} \phi (y)=s\phi (p(y)), \quad y\in {\mathbb {R}}_+, \end{aligned}$$

and, inverting, we obtain the conjugacy

$$\begin{aligned} p(y) = \phi ^{-1}\big (\rho \phi (y)\big ). \end{aligned}$$

Hence

$$\begin{aligned} x_{n,n} = p^n(x/\rho ^n) = \phi ^{-1}\big (\rho ^n \phi (x/\rho ^n)\big )\xrightarrow [n\rightarrow \infty ]{} \phi ^{-1}\big (x \phi '(0+)\big ) = \phi ^{-1}(x). \end{aligned}$$

In particular, (20) and, therefore also (19), hold. $\square$

#### Properties of ${\mathbf {f}}(\cdot )$

**Fig. 3:**
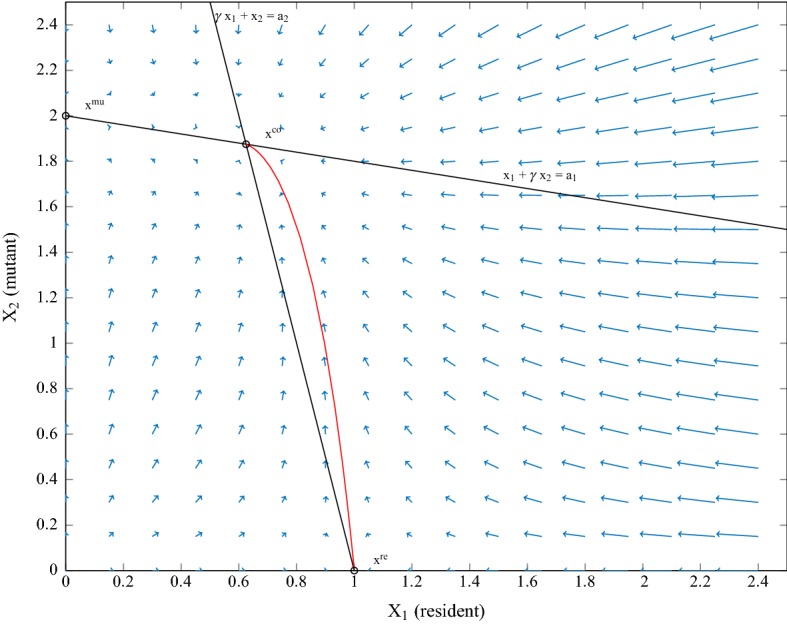
The phase-portrait of the deterministic system (3); the trajectory from a small vicinity of the resident equilibrium ${\mathbf {x}}^{\mathrm {re}}$ to the coexistence equilibrium ${\mathbf {x}}^{\mathrm {co}}$ is depicted in red (colour figure online)

Let us summarize some relevant properties of the function ${\mathbf {f}}(\cdot )$, which governs the deterministic dynamics in (3). Since $f_1({\mathbf {x}})-x_1$ and $f_2({\mathbf {x}})-x_2$ change signs across the lines $x_1+\gamma x_2 =a_1$ and $\gamma x_1 + x_2 =a_2$ respectively, as shown at the phase portrait (Fig. 3), the coexistence equilibrium ${\mathbf {x}}^{\mathrm {co}}$ is globally stable. The local behaviour around the unstable fixed point ${\mathbf {x}}^{\mathrm {re}}=(a_1,0)$ is determined the Jacobian of ${\mathbf {f}}(\cdot )$ at ${\mathbf {x}}^{\mathrm {re}}$,

22 $$\begin{aligned} A:= D {\mathbf {f}}( {\mathbf {x}}^{\mathrm {re}}) =\begin{pmatrix} \frac{1}{2} &{}\quad -\frac{ \gamma }{2}\\ 0 &{} \quad \rho \end{pmatrix}, \ \text {where}\;\;\rho = m_{2}({\mathbf {x}}^{\mathrm {re}})=\frac{2 a_2 }{ a_2+\gamma a_1 }. \end{aligned}$$

To study perturbations around ${\mathbf {x}}^{\mathrm {re}}$ it will be convenient to consider the translation

23 $$\begin{aligned} {\mathbf {g}}({\mathbf {x}}):= {\mathbf {f}}({\mathbf {x}}^{\mathrm {re}}+{\mathbf {x}})-{\mathbf {x}}^{\mathrm {re}}, \end{aligned}$$

with ${\mathbf {g}}({\mathbf {0}})={\mathbf {0}}$ and Jacobian $D\,{\mathbf {g}}({\mathbf {0}})=A$. In particular, existence of the limit (13) follows from that of $\lim _{n\rightarrow \infty } {\mathbf {g}}^n({\mathbf {x}})$.

Note that for $x_1\in [x^{\mathrm {co}}_1, \infty )$ and $x_2 \in [0,x^{\mathrm {co}}_2]$, formulas (4) for the entries of the function ${\mathbf {f}}(\cdot )$ and the configuration of the fixed points (7) imply

$$\begin{aligned} f_1( {\mathbf {x}}) = \frac{2x_1a_1}{a_1+x_1+\gamma x_2} \ge \frac{2x^{\mathrm {co}}_1a_1}{a_1+x^{\mathrm {co}}_1+\gamma x^{\mathrm {co}}_2}=x^{\mathrm {co}}_1 \end{aligned}$$

and

$$\begin{aligned} f_2({\mathbf {x}}) = \frac{2x_2a_2}{a_2+\gamma x_1+ x_2} \le \frac{2x_2^{\mathrm {co}}a_2}{a_2+\gamma x_1^{\mathrm {co}}+ x_2^{\mathrm {co}}}=x_2^{\mathrm {co}}. \end{aligned}$$

Hence the subset ${\widetilde{E}} := [x^{\mathrm {co}}_1, \infty )\times [0,x^{\mathrm {co}}_2]\subset {\mathbb {R}}^2_+$ is forward invariant under ${\mathbf {f}}(\cdot )$, namely, ${\mathbf {f}}({\widetilde{E}})\subseteq {\widetilde{E}}$. Then by (23) the subset

$$\begin{aligned} E = \left[ x^{\mathrm {co}}_1-a_1, \infty \right) \times \left[ 0,x^{\mathrm {co}}_2\right] \subset {\mathbb {R}}\times {\mathbb {R}}_+ \end{aligned}$$

is forward invariant under ${\mathbf {g}}(\cdot )$.

In what follows, $\Vert \cdot \Vert$ stands for the $\ell _\infty$ norm for vectors and the corresponding operator norm for matrices. In particular, the matrix *A* defined in (22) satisfies $\Vert A\Vert = \rho >1$. The linear subspace $E_0 =\{{\mathbf {x}}\in {\mathbb {R}}^2: x_2=0\}$ is invariant under *A* and

24 $$\begin{aligned} \sup _{{\mathbf {x}}\in E_0} \frac{\Vert A{\mathbf {x}}\Vert }{\Vert {\mathbf {x}}\Vert }=\frac{1}{2}. \end{aligned}$$

Below *C*, $C_1$, etc. stand for constants, which depend only on $a_1$, $a_2$ and $\gamma$ and whose values may change from line to line.

The first coordinate of ${\mathbf {g}}(\cdot )$ can be written as

$$\begin{aligned} g_1({\mathbf {x}})&= \frac{2 a_1 (a_1+x_1) }{2a_1 +x_1+\gamma x_2}-a_1 = \frac{ a_1x_1 }{2a_1 +x_1+\gamma x_2} - \frac{ a_1\gamma x_2}{2a_1 +x_1+\gamma x_2} \\&= \frac{1}{2} x_1 \left( 1 - \frac{ x_1+\gamma x_2 }{2a_1 +x_1+\gamma x_2} \right) -\frac{\gamma }{2} x_2\left( 1- \frac{ x_1+\gamma x_2}{2a_1 +x_1+\gamma x_2} \right) , \end{aligned}$$

and, similarly,

$$\begin{aligned} g_2({\mathbf {x}}) = \rho x_2\left( 1- \frac{ x_2 +\gamma x_1}{a_2 +\gamma a_1+ x_2+\gamma x_1 }\right) . \end{aligned}$$

Hence ${\mathbf {g}}({\mathbf {x}})$ has the form

25 $$\begin{aligned} {\mathbf {g}}({\mathbf {x}}) = \big (I-B({\mathbf {x}})\big )A {\mathbf {x}}\end{aligned}$$

where matrix $B({\mathbf {x}})$ satisfies the bound

26 $$\begin{aligned} \Vert B({\mathbf {x}})\Vert \le C \Vert {\mathbf {x}}\Vert , \quad {\mathbf {x}}\in E \end{aligned}$$

with a constant *C*. Similar calculation also shows that for ${\mathbf {x}},{\mathbf {y}}\in E$

27 $$\begin{aligned} g({\mathbf {x}})-g({\mathbf {y}}) = \big (A+F({\mathbf {x}},{\mathbf {y}})\big )({\mathbf {x}}-{\mathbf {y}}) \end{aligned}$$

where matrix $F({\mathbf {x}},{\mathbf {y}})$ satisfies

28 $$\begin{aligned} \Vert F({\mathbf {x}},{\mathbf {y}})\Vert \le C \big (\Vert {\mathbf {x}}\Vert \vee \Vert {\mathbf {y}}\Vert \big ). \end{aligned}$$

These formulas and the bound from Lemma 1 give the following growth estimate.

##### Lemma 2

For any ${\mathbf {x}}\in {\mathbb {R}}\times {\mathbb {R}}_+$ and all *n* large enough,

29 $$\begin{aligned} \big \Vert {\mathbf {g}}^m({\mathbf {x}}/\rho ^n)\big \Vert \le \psi (\Vert {\mathbf {x}}\Vert ) \rho ^{m-n}, \quad m=1,\dots ,n \end{aligned}$$

with a finite function $\psi (r)$, $r\ge 0$.

##### Proof

For any ${\mathbf {x}}\in {\mathbb {R}}\times {\mathbb {R}}_+$ and all *n* large enough ${\mathbf {x}}/\rho ^n\in E$ and, since *E* is invariant, ${\mathbf {g}}^m({\mathbf {x}}/\rho ^n)\in E$ for all *m*. Hence by (25), the sequence ${\mathbf {x}}_{m,n}={\mathbf {g}}^m({\mathbf {x}}/\rho ^n)$ satisfies

$$\begin{aligned} \Vert {\mathbf {x}}_{m+1,n}\Vert&= \Vert {\mathbf {g}}\big ({\mathbf {x}}_{m,n}\big ) \Vert = \big \Vert \big (I-B({\mathbf {x}}_{m,n})\big )A {\mathbf {x}}_{m,n} \big \Vert \\&\quad \le \Vert I-B({\mathbf {x}}_{m,n})\Vert \Vert A \Vert \Vert {\mathbf {x}}_{m,n}\Vert \le \rho \Vert {\mathbf {x}}_{m,n}\Vert (1+C \Vert {\mathbf {x}}_{m,n}\Vert ). \end{aligned}$$

By induction $\Vert {\mathbf {x}}_{m,n}\Vert \le x_{m,n}$, where $x_{m,n}$ solves (18) subject to $x_{0,n}=\Vert {\mathbf {x}}\Vert /\rho ^n$, and the claim follows from Lemma 1.$\square$

#### Proof of Theorem 2

We will argue that the increments of ${\mathbf {g}}^n({\mathbf {x}}/\rho ^n)$ are absolutely summable, uniformly over compacts in ${\mathbb {R}}\times {\mathbb {R}}_+$. Let *n* be large enough so that ${\mathbf {x}}/\rho ^n\in E$ and therefore, by invariance, ${\mathbf {g}}^{m}({\mathbf {x}}/\rho ^n)\in E$ for all $m\ge 1$. Consider the array

$$\begin{aligned} {\mathbf {g}}^{m}({\mathbf {x}}/\rho ^{n+1}) - {\mathbf {g}}^{m-1}({\mathbf {x}}/\rho ^n), \quad m=1,\dots ,n. \end{aligned}$$

For $m=1$, due to (25),

$$\begin{aligned} {\mathbf {g}}({\mathbf {x}}/\rho ^{n+1})- {\mathbf {x}}/\rho ^n&= A {\mathbf {x}}/\rho ^{n+1}- {\mathbf {x}}/\rho ^n-B({\mathbf {x}}/\rho ^{n+1})A {\mathbf {x}}/\rho ^{n+1} \\&= \rho ^{-n}\big ( A/\rho -I\big ){\mathbf {x}}+ \rho ^{-2n} {\mathbf {v}}_n =: \rho ^{-n} {\mathbf {u}} + \rho ^{-2n} {\mathbf {v}}_n, \end{aligned}$$

where ${\mathbf {u}}\in E_0$ and, in view of (26), ${\mathbf {v}}_n$ is a sequence of vectors, whose norm is bounded uniformly in *n*. Both vectors ${\mathbf {u}}$ and ${\mathbf {v}}_n$ depend continuously on ${\mathbf {x}}$, which is omitted from the notations. For $m\ge 1$, (27) implies

$$\begin{aligned}&{\mathbf {g}}^{m+1}({\mathbf {x}}/\rho ^{n+1}) - {\mathbf {g}}^{m}({\mathbf {x}}/\rho ^n) = {\mathbf {g}}\circ {\mathbf {g}}^{m} ({\mathbf {x}}/\rho ^{n+1}) - {\mathbf {g}}\circ {\mathbf {g}}^{m-1}({\mathbf {x}}/\rho ^n) \\&\quad = \Big (A + F\big ({\mathbf {g}}^m ({\mathbf {x}}/\rho ^{n+1}),{\mathbf {g}}^{m-1}({\mathbf {x}}/\rho ^n)\big )\Big )\Big ({\mathbf {g}}^m ({\mathbf {x}}/\rho ^{n+1})-{\mathbf {g}}^{m-1}({\mathbf {x}}/\rho ^n)\Big ) \end{aligned}$$

and, letting $F_{m,n} := F\big ({\mathbf {g}}^m ({\mathbf {x}}/\rho ^{n+1}),{\mathbf {g}}^{m-1}({\mathbf {x}}/\rho ^n)\big )$ , we get

30 $$\begin{aligned} {\mathbf {g}}^{n+1}({\mathbf {x}}/\rho ^{n+1}) - {\mathbf {g}}^n({\mathbf {x}}/\rho ^n) = \left\{ \prod _{m=1}^n \Big (A + F_{m,n}\Big ) \right\} \big (\rho ^{-n} {\mathbf {u}} + \rho ^{-2n} {\mathbf {v}}_{n}\big ). \end{aligned}$$

Since $\Vert A\Vert =\rho$, by virtue of (29) and (28)

31 $$\begin{aligned} \begin{aligned}&\left\| \left\{ \prod _{m=1}^n \big (A + F_{m,n}\big ) \right\} \rho ^{-2n} {\mathbf {v}}_{n} \right\| \le \rho ^{-2n}\Vert {\mathbf {v}}_{n}\Vert \prod _{m=1}^n \Big (\big \Vert A \big \Vert + \big \Vert F_{m,n} \big \Vert \Big ) \\&\quad \le \rho ^{-2n} C_1 \prod _{m=1}^n \Big (\rho + C \big (\Vert {\mathbf {g}}^m ({\mathbf {x}}/\rho ^{n+1})\Vert \vee \Vert {\mathbf {g}}^{m-1}({\mathbf {x}}/\rho ^n) \Vert \big )\Big ) \\&\quad \le \rho ^{-2n}C_1 \prod _{m=1}^n \big (\rho + C_2 \rho ^{m-n}\big )= \rho ^{- n}C_1 \prod _{m=1}^n \big (1+ C_2 \rho ^{m-n-1}\big )\le C_3\rho ^{- n}, \end{aligned} \end{aligned}$$

where constants $C_j$’s depend continuously on $\Vert x\Vert$.

Let us now bound the remaining term in (30). To this end, observe that

$$\begin{aligned} \prod _{m=1}^n\Big (A + F_{m,n}\Big )&= \prod _{m=2}^n\Big (A + F_{m,n}\Big ) F_{1,n} \\&\quad + \prod _{m=3}^n\Big (A + F_{m,n}\Big ) F_{2,n} A \\&\quad + \prod _{m=4}^n\Big (A + F_{m,n}\Big ) F_{3,n} A^2 + \cdots \\&\quad + \Big (A + F_{n,n}\Big )F_{n-1,n}A^{n-2} + F_{n,n} A^{n-1} + A^n. \end{aligned}$$

Since ${\mathbf {u}}\in E_0$ and $E_0$ is invariant under *A*, we have $\Vert A^k{\mathbf {u}}\Vert \le (1/2)^k\Vert {\mathbf {u}}\Vert$ due to (24). Therefore for all $k=0,\dots ,n-2$

$$\begin{aligned}&\Big \Vert \prod _{m=k+2}^n\Big (A + F_{m,n}\Big ) F_{k+1,n} A^{k} {\mathbf {u}} \Big \Vert \\&\quad \le (1/2)^k\Vert {\mathbf {u}}\Vert \Big \Vert \prod _{m=k+2}^n\Big (A + F_{m,n}\Big ) F_{k+1,n} \Big \Vert \\&\quad \le C_1 (1/2)^k \Vert F_{k+1,n}\Vert \prod _{m=k+2}^n\Big (\Vert A\Vert + \Vert F_{m,n}\Vert \Big ) \\&\quad \le C_2 (1/2)^k\Vert {\mathbf {x}}\Vert \rho ^{k-n} \prod _{m=k+2}^n\Big (\rho + C \Vert {\mathbf {x}}\Vert \rho ^{m-n}\Big ) \\&\quad \le C_3 (1/2)^k \prod _{m=k+2}^n\Big (1 + C_4\rho ^{m-n}\Big ) \\&\quad \le C_3 (1/2)^k \exp \left( C_4\sum _{m=k+2}^n \rho ^{m-n} \right) \le C_5 (1/2)^k , \end{aligned}$$

where all $C_j$’s depend continuously on ${\mathbf {x}}$. Consequently

$$\begin{aligned} \left\| \left\{ \prod _{m=1}^n \Big (A + F_{m,n}\Big ) \right\} \rho ^{-n} {\mathbf {u}} \right\| \le C_6 \rho ^{-n}. \end{aligned}$$

Plugging this and (31) into (30) yields

$$\begin{aligned} \big \Vert {\mathbf {g}}^{n+1}({\mathbf {x}}/\rho ^{n+1}) - {\mathbf {g}}^n({\mathbf {x}}/\rho ^n)\big \Vert \le C_7 \rho ^{-n}, \end{aligned}$$

where $C_7$ depends continuously on ${\mathbf {x}}$. This implies that ${\mathbf {g}}^n({\mathbf {x}}/\rho ^n)$ converges as $n\rightarrow \infty$, uniformly on compacts. Existence of the limit ${\mathbf {H}}({\mathbf {x}})$ in (13) now follows from (23), and Theorem 2 is proved. $\square$

### The main approximation

In this section we construct the random variable *W* and prove convergence (11). To this end, let *U*(*n*, *j*) and *V*(*n*, *j*) be i.i.d. random variables distributed uniformly over the unit interval [0, 1] and define $\xi _1(n,j)$ and $\xi _2(n,j)$ in (1) as

32 $$\begin{aligned} \begin{aligned} \xi _1(n,j)&= 2\cdot {{\mathbf {1}}}\bigg \{U(n,j) \le \frac{a_1K}{a_1K+Z_1(n)+\gamma Z_2(n)}\bigg \}, \\ \xi _2(n,j)&= 2\cdot {{\mathbf {1}}}\bigg \{V(n,j) \le \frac{a_2K}{a_2K+\gamma Z_1(n)+ Z_2(n)}\bigg \}. \end{aligned} \end{aligned}$$

Define Galton–Watson branching process ${\mathbf {Y}}(n)$ with components

$$\begin{aligned} Y_1(n+1)&= \sum _{j=1}^{Y_1(n) } 2\cdot {{\mathbf {1}}}\bigg \{U(n,j) \le \frac{1}{2}\bigg \}, \\ Y_2(n+1)&= \sum _{j=1}^{Y_2(n)} 2\cdot {{\mathbf {1}}}\bigg \{V(n,j) \le \frac{1}{2} \rho \bigg \}, \end{aligned}$$

subject to $Y_1(0) = [a_1 K]$ and $Y_2(0) = 1$, and the corresponding density process $\overline{{\mathbf {Y}}}(n)={\mathbf {Y}}(n)/K$. Note that $Y_2(n)$ coincides in distribution with the process defined in (12) and

33 $$\begin{aligned} \rho ^{-n} Y_2(n)\xrightarrow [n\rightarrow \infty ]{{\mathbb {P}}}W. \end{aligned}$$

Finally, for a fixed constant $c\in (\frac{1}{2},1]$ define

$$\begin{aligned} n_c(K) := \left[ \log _\rho K^c\right] = \left[ \frac{c}{\log \rho }\log K\right] . \end{aligned}$$

In particular, $n_1(K) = [b\log K] = \big [ \log _\rho K\big ]$, cf. Theorem 1.

As explained in Sect. 3.1, the limit (11) follows from (14) and (15).

#### Proof of (15)

Since ${\mathbb {E}}Y_1(n)=[a_1 K]$ and ${\mathrm {Var}}\big (Y_1(n)\big ) \le n a_1K$ we have

34 $$\begin{aligned} {\mathbb {E}}\big ( Y_1(n_c)-a_1 K \big )^2 \le a_1 K \log _\rho K^c \end{aligned}$$

and hence for any $c\in (\frac{1}{2},1)$,

35 $$\begin{aligned} \rho ^{-n_c} \big ( Y_1(n_c)- a_1 K \big )\xrightarrow [K\rightarrow \infty ]{{\mathbb {P}}} 0. \end{aligned}$$

This along with (33) implies (17), and in view of representation (16), the limit in (15) follows by the continuous mapping theorem and the uniform convergence in (13). $\square$

#### Proof of (14)

Since

$$\begin{aligned} \big \Vert \overline{{\mathbf {Z}}}(n_1) - {\widetilde{{\mathbf {Z}}}}(n_1)\big \Vert \le \, \big \Vert \overline{{\mathbf {Z}}}(n_1)-{\mathbf {f}}^{n_1-n_c} (\overline{{\mathbf {Z}}}(n_c))\big \Vert + \big \Vert {\mathbf {f}}^{n_1-n_c} (\overline{{\mathbf {Z}}}(n_c))- {\mathbf {f}}^{n_1-n_c}(\overline{{\mathbf {Y}}}(n_c))\big \Vert \end{aligned}$$

it suffices to prove that

36 $$\begin{aligned} \big \Vert \overline{{\mathbf {Z}}}(n_1)-{\mathbf {f}}^{n_1-n_c} \big (\overline{{\mathbf {Z}}}(n_c)\big )\big \Vert \xrightarrow [K\rightarrow \infty ]{{\mathbb {P}}} 0 \end{aligned}$$

and

37 $$\begin{aligned} \big \Vert {\mathbf {f}}^{n_1-n_c} \big (\overline{{\mathbf {Z}}}(n_c)\big )- {\mathbf {f}}^{n_1-n_c}\big (\overline{{\mathbf {Y}}}(n_c)\big )\big \Vert \xrightarrow [K\rightarrow \infty ]{{\mathbb {P}}} 0. \end{aligned}$$

Let us first prove (36). Recall that the density process ${\mathbf {X}}(n)=\overline{{\mathbf {Z}}}(n)$ solves (6),

$$\begin{aligned} \overline{{\mathbf {Z}}}(n) = {\mathbf {f}}\big (\overline{{\mathbf {Z}}}(n-1)\big ) +\frac{1}{\sqrt{K}} \varvec{\eta }(n), \end{aligned}$$

and hence the difference ${\varvec{\delta }}(n) := \overline{{\mathbf {Z}}}(n)-{\mathbf {f}}^{n-n_c} (\overline{{\mathbf {Z}}}(n_c))$ satisfies

$$\begin{aligned} {\varvec{\delta }}(n) = {\mathbf {f}}\big (\overline{{\mathbf {Z}}}(n-1)\big ) -{\mathbf {f}}\big (\overline{{\mathbf {Z}}}(n-1)-{\varvec{\delta }}(n-1)\big ) + \frac{1}{\sqrt{K}} \varvec{\eta }(n), \quad n> n_c \end{aligned}$$

subject to ${\varvec{\delta }}(n_c)=0$. A direct calculation shows that the Jacobian of ${\mathbf {f}}(\cdot )$ is bounded

$$\begin{aligned} {\widetilde{\rho }} := \sup _{{\mathbf {x}}\in {\mathbb {R}}^2_+}\Vert D\, {\mathbf {f}}({\mathbf {x}})\Vert _\infty \in (\rho , 2], \end{aligned}$$

Hence ${\mathbf {f}}(\cdot )$ is ${\widetilde{\rho }}$-Lipschitz on ${\mathbb {R}}_+^2$ with respect to $\ell _\infty$ norm and

$$\begin{aligned} \Vert {\varvec{\delta }}(n)\Vert \le {\widetilde{\rho }}\, \Vert {\varvec{\delta }}(n-1)\Vert + \frac{1}{\sqrt{K}} \Vert \varvec{\eta }(n)\Vert . \end{aligned}$$

Let $$\beta := \log _\rho {\widetilde{\rho }} > 1$$, then

$$\begin{aligned} {\mathbb {E}}\Vert {\varvec{\delta }}(n_1)\Vert&\le \frac{1}{\sqrt{K}} \sum _{j=n_c}^{n_1} \widetilde{\rho }^{\, n_1-j} {\mathbb {E}}\Vert \varvec{\eta }(j)\Vert \\&\le \frac{1}{\sqrt{K}} (n_1-n_c){\widetilde{\rho }}^{n_1-n_c} \sup _{j\le n_1}{\mathbb {E}}\Vert \varvec{\eta }(j)\Vert \\&\le C K^{(1-c)\beta -\frac{1}{2}}\log _{\rho }K^{1-c} \rightarrow 0, \end{aligned}$$

where convergence holds if *c* is chosen close enough to 1. This proves (36).

To check (37), write

$$\begin{aligned}&\Big \Vert {\mathbf {f}}^{n_1-n_c} \big (\overline{{\mathbf {Z}}}(n_c)\big )- {\mathbf {f}}^{n_1-n_c}\big (\overline{{\mathbf {Y}}}(n_c)\big )\Big \Vert \\&\quad = \Big \Vert {\mathbf {f}}^{n_1-n_c} \Big ({\mathbf {x}}^{\mathrm {re}}+\rho ^{-\{\log _\rho K\}}\rho ^{-(n_1-n_c)} \rho ^{-n_c}({\mathbf {Z}}(n_c)-K{\mathbf {x}}^{\mathrm {re}}) \Big ) \\&\qquad -{\mathbf {f}}^{n_1-n_c} \Big ({\mathbf {x}}^{\mathrm {re}}+\rho ^{-\{\log _\rho K\}}\rho ^{-(n_1-n_c)} \rho ^{-n_c}({\mathbf {Y}}(n_c)-K{\mathbf {x}}^{\mathrm {re}}) \Big ) \Big \Vert . \end{aligned}$$

Since, by (33) and (35), the sequence $\rho ^{-n_c}({\mathbf {Y}}(n_c)-K{\mathbf {x}}^{\mathrm {re}})$ converges to (0, *W*) in probability and, by Theorem 2, the sequence ${\mathbf {f}}^n({\mathbf {x}}^{\mathrm {re}}+{\mathbf {x}}/\rho ^n)$ converges uniformly on compacts to ${\mathbf {H}}({\mathbf {x}})$, it suffices to show that

$$\begin{aligned} \rho ^{-n_c} \Vert {\mathbf {Z}}(n_c)-{\mathbf {Y}}(n_c)\Vert \xrightarrow [K\rightarrow \infty ]{{\mathbb {P}}}0, \end{aligned}$$

that is,

38 $$\begin{aligned} K^{-c}\big |Z_j(n_c)-Y_j(n_c)\big | \rightarrow 0,\quad j=1,2, \end{aligned}$$

where $c\in (\frac{1}{2}, 1)$ has been already fixed in the previous calculations. We prove (38) for $j=2$, omitting the similar proof for the case $j=1$.

To this end, choose arbitrary constants

$$\begin{aligned} \alpha _{1\ell }, \alpha _{1u}, \alpha _2 \in (c,1)\subset (\tfrac{1}{2} ,1), \end{aligned}$$

and, using the same random variables *U*(*n*, *j*) and *V*(*n*, *j*) as in (32), define two additional auxiliary Galton–Watson branching processes ${\mathbf {L}}(n)$ and ${\mathbf {U}}(n)$ with the entries

$$\begin{aligned}&L_1(n) = \sum _{j=1}^{L_1(n-1)}2\cdot {{\mathbf {1}}}\bigg \{U(n,j) \le \frac{1}{2} r^-_K \bigg \}, \quad L_1(0)=[a_1K], \\&U_1(n) = \sum _{j=1}^{U_1(n-1)}2\cdot {{\mathbf {1}}}\bigg \{U(n,j) \le \frac{1}{2} r^+_K \bigg \}, \quad U_1(0)=[a_1K], \end{aligned}$$

where

$$\begin{aligned}&r^-_K := \frac{2a_1}{a_1+ a_1(1+ K^{\alpha _{1u}-1})+ \gamma K^{\alpha _2-1}} < 1 \quad \text {and} \\&r^+_K := \frac{2a_1}{a_1+ a_1(1- K^{\alpha _{1\ell }-1}) } > 1, \end{aligned}$$

and

$$\begin{aligned}&L_2(n) = \sum _{j=1}^{L_2(n-1)}2\cdot {{\mathbf {1}}}\bigg \{V(n,j) \le \frac{1}{2} \rho ^-_K \bigg \}, \quad L_2(0)=1, \\&U_2(n) = \sum _{j=1}^{U_2(n-1)}2\cdot {{\mathbf {1}}}\bigg \{V(n,j) \le \frac{1}{2} \rho ^+_K \bigg \}, \quad U_2(0) =1, \end{aligned}$$

where

$$\begin{aligned} \rho ^-_K= & {} \frac{2 a_2}{a_2+\gamma a_1 (1+K^{\alpha _{1u}-1}) +K^{\alpha _2-1}}< \rho \quad \text {and} \\ \rho ^+_K= & {} \frac{ 2a_2}{a_2+\gamma a_1 (1-K^{\alpha _{1\ell }-1})}>\rho . \end{aligned}$$

Define the exit times

39 $$\begin{aligned} \begin{aligned} \tau ^{1\ell }&= \min \big \{n: Z_1(n) \le a_1 (K-K^{\alpha _{1\ell } }) \big \}, \\ \tau ^{1u}&= \min \big \{n: Z_1(n) \ge a_1 (K+K^{\alpha _{1u} }) \big \}, \\ \tau ^2&= \min \big \{n: Z_2(n)\ge K^{\alpha _2 } \big \}. \end{aligned} \end{aligned}$$

The random variable $\tau = \tau ^{1\ell } \wedge \tau ^{1u} \wedge \tau ^2$ is a coupling time for the above processes, in the sense that

$$\begin{aligned} {\mathbb {P}}\big (L_2(n) \le Y_2(n)\le U_2(n)\big )=1, \end{aligned}$$

and

$$\begin{aligned} \{\tau \ge n\}\subseteq \big \{L_2(n) \le Z_2(n)\le U_2(n)\big \}. \end{aligned}$$

Hence

$$\begin{aligned} \big |Z_2(n) - Y_2(n)\big |\le \big (U_2(n) - L_2(n)\big ){1}_{\{\tau \ge n\}} + \big |Z_2(n) - Y_2(n)\big |{1}_{\{\tau < n\}}. \end{aligned}$$

Convergence (38) for $j=2$ holds, if we show that

40 $$\begin{aligned} K^{-c}\big (U_2(n_c) - L_2(n_c)\big )\xrightarrow [n\rightarrow \infty ]{{\mathbb {P}}} 0, \end{aligned}$$

and

41 $$\begin{aligned} {\mathbb {P}}(\tau < n_c)\xrightarrow [K\rightarrow \infty ]{}0, \end{aligned}$$

since $\big \{K^{-c}\big |Z_2(n) - Y_2(n)\big |{1}_{\{\tau<n\}}\ge \varepsilon \big \}\subseteq \{\tau < n\}$ for any $\varepsilon >0$.

The limit in (40) holds, since

$$\begin{aligned}&K^{-c} {\mathbb {E}}\big (U_2(n_c)- L_2(n_c)\big ) \le \left( \rho ^+_K/\rho \right) ^{n_c}-\left( \rho ^-_K/\rho \right) ^{n_c} \le \frac{\rho ^+_K-\rho ^-_K}{\rho } \big (\rho ^+_K/\rho \big )^{n_c}n_c \\&\quad \le C\, \Big |K^{\alpha _{1\ell }-1}+K^{\alpha _{1u}-1}+K^{\alpha _2-1}\Big | \big (1+ O(K^{\alpha _{1\ell }-1})\big )^{\log _\rho K^c}\log _\rho K^c \xrightarrow [K\rightarrow \infty ]{}0. \end{aligned}$$

It is left to prove (41). Since

$$\begin{aligned} {\mathbb {P}}(\tau< n_c) \le {\mathbb {P}}\left( \tau ^{1\ell }<n_c\right) + {\mathbb {P}}\left( \tau ^{1u}<n_c\right) + {\mathbb {P}}\left( \tau ^2 <n_c\right) , \end{aligned}$$

it suffices to check that each of the exit times, c.f. (39),

$$\begin{aligned} \tau ^{1\ell }(\alpha )&= \min \big \{n: Z_1(n) \le a_1 (K-K^{\alpha }) \big \},\\ \tau ^{1u}(\alpha )&= \min \big \{n: Z_1(n) \ge a_1 (K+K^{\alpha }) \big \}, \\ \tau ^2 (\alpha )&= \min \big \{n: Z_2(n)\ge K^{\alpha } \big \}, \end{aligned}$$

satisfy

42a $$\begin{aligned}&{\mathbb {P}}\big (\tau ^{1\ell }(\alpha )\le n_c \big )\xrightarrow [K\rightarrow \infty ]{} 0, \end{aligned}$$

42b $$\begin{aligned}&{\mathbb {P}}\big (\tau ^{1u}(\alpha )\le n_c \big )\xrightarrow [K\rightarrow \infty ]{} 0, \end{aligned}$$

42c $$\begin{aligned}&{\mathbb {P}}\big (\tau ^{2}(\alpha )\le n_c \big )\xrightarrow [K\rightarrow \infty ]{} 0, \end{aligned}$$

for *any*$\alpha \in ( c , 1)$. To this end, we can reuse the auxiliary processes ${\mathbf {L}}(n)$ and ${\mathbf {U}}(n)$, defined above, with appropriately chosen parameters $\alpha _{1\ell }$, $\alpha _{1u}$ and $\alpha _2$. Define exit times

$$\begin{aligned} \sigma ^2&= \min \{n: U_2(n)\ge K^{\alpha _2} \}, \\ \sigma ^{1u}&= \min \{n: U_1(n) \ge a_1(K+K^{\alpha _{1u}})\}, \\ \sigma ^{1\ell }&= \min \{n: L_1(n) \le a_1(K-K^{\alpha _{1\ell }})\}. \end{aligned}$$

To prove (42c), we can choose $\alpha _{1\ell } = \alpha _{1u}=\alpha _2 = \alpha$. By construction,

$$\begin{aligned} \left\{ \tau ^2(\alpha )<n_c\right\} \subseteq \left\{ \sigma ^2< n_c\right\} , \end{aligned}$$

and therefore

$$\begin{aligned}&{\mathbb {P}}\left( \tau ^2(\alpha )< n_c\right) \le {\mathbb {P}}\left( \sigma ^2<n_c\right) = {\mathbb {P}}\left( \max _{n\le n_c}U_2(n)\ge K^{\alpha _2}\right) \\&\quad ={\mathbb {P}}\left( \left( \rho _K^+\right) ^{-n_c}\max _{n\le n_c}U_2(n)\ge \left( \rho _K^+\right) ^{-n_c}K^{\alpha _2}\right) \\&\quad \le {\mathbb {P}}\left( \max _{n\le n_c}\left( \rho _K^+\right) ^{-n} U_2(n)\ge \left( \rho _K^+\right) ^{-n_c}K^{\alpha _2}\right) \\&\quad {\mathop {\le }\limits ^{\dagger }} \left( \rho _K^+\right) ^{n_c}K^{-\alpha _2} \le C K^{c-\alpha _2}\xrightarrow [K\rightarrow \infty ]{} 0, \end{aligned}$$

where $\dagger$ holds by Doob’s inequality (Shiryaev 1996, Theorem VII.3.3, p. 493, eq. (11)), applied to the martingale $(\rho _K^+)^{-n} U_2(n)$.

To prove (42b), let us choose $c< \alpha _{1u}=\alpha _2 < \alpha _{1\ell }=\alpha$. By construction,

$$\begin{aligned} \left\{ \tau ^{1u}(\alpha )< n_c\right\} \subseteq \left\{ \sigma ^{1u}<n_c\right\} , \end{aligned}$$

and since the process $[a_1K]-L_1(n)$ is a submartingale,

$$\begin{aligned}&{\mathbb {P}}\left( \tau ^{1\ell }(\alpha )<n_c\right) \le {\mathbb {P}}\left( \sigma ^{1\ell }<n_c\right) = {\mathbb {P}}\left( \min _{n\le n_c}L_1(n)\le a_1 (K-K^{\alpha _{1\ell }})\right) \\&\quad = {\mathbb {P}}\left( \max _{n\le n_c}\big ([a_1K] -L_1(n)\big )\ge a_1 K^{\alpha _{1\ell }} -1\right) \\&\quad {\mathop {\le }\limits ^{\dagger }} C K^{-\alpha _{1\ell }}{\mathbb {E}}\big |[a_1K]-L_1(n_c)\big | \\&\quad {\mathop {\le }\limits ^{\ddagger }} C K^{-\alpha _{1\ell }} \big | [a_1K] -{\mathbb {E}}L_1(n_c)\big | + C K^{-\alpha _{1\ell }}\sqrt{{\mathrm {Var}}\big ( L_1(n_c)\big )}, \end{aligned}$$

where $\dagger$ is another variant of Doob’s inequality (Shiryaev 1996, Theorem VII.3.1, p. 492, eq. (1)) and $\ddagger$ holds by the Jensen inequality. The first term satisfies

$$\begin{aligned}&K^{-\alpha _{1\ell }} \big |[a_1K]-{\mathbb {E}}L_1(n_c)\big | = K^{-\alpha _{1\ell }} [a_1K] \big |1- \left( r^-_K\right) ^{n_c}\big | \\&\quad \le C_1 K^{1-\alpha _{1\ell }} \Big |1- \Big (1- K^{\alpha _{1u}-1}- \frac{\gamma }{a_1}K^{\alpha _2-1}\Big )^{n_c}\Big | \\&\quad \le C_2 K^{1-\alpha _{1\ell }} \big (K^{\alpha _{1u}-1}+K^{\alpha _2-1}\big ) n_c \\&\quad \le C_3 \big (K^{\alpha _{1u}-\alpha _{1\ell }} + K^{\alpha _2-\alpha _{1\ell }} \big ) \log _\rho K \xrightarrow [K\rightarrow \infty ]{}0, \end{aligned}$$

where the convergence holds by the choice $\alpha _{1u}=\alpha _2<\alpha _{1\ell }$. The second term satisfies

$$\begin{aligned} K^{-\alpha _{1\ell }}\sqrt{{\mathrm {Var}}( L_1(n_c))} \le K^{-\alpha _{1\ell }}\sqrt{ a_1 K n_c (r^-_K)^{n_c}}\le C K^{\frac{1}{2}-\alpha _{1\ell }}\log _\rho K \xrightarrow [K\rightarrow \infty ]{}0. \end{aligned}$$

Finally, to prove (42a), let us choose $c< \alpha _2 = \alpha _{1\ell } < \alpha _{1u}=\alpha$, then

$$\begin{aligned}&{\mathbb {P}}\big (\tau ^{1u}(\alpha )< n_c \big ) \le {\mathbb {P}}\left( \sigma ^{1u}< n_c\right) = {\mathbb {P}}\left( \max _{n\le n_c}U_1(n) \ge a_1 \left( K+K^{\alpha _{1u}}\right) \right) \\&\quad \le {\mathbb {P}}\left( \max _{n\le n_c}\big (\left( r^+_K\right) ^{-n} U_1(n) -[a_1K]\big )\ge [a_1K] \left( \left( r^+_K\right) ^{-n_c}-1\right) +a_1\left( r^+_K\right) ^{-n_c}K^{\alpha _{1u}} \right) \\&\quad {\mathop {\le }\limits ^{\dagger }} C_1 K^{-\alpha _{1u}} {\mathbb {E}}\big |\left( r^+_K\right) ^{-n_c} U_1(n_c) -[a_1K]\big | \le C_2 K^{-\alpha _{1u}} \sqrt{{\mathrm {Var}}(U_1(n_c))} \\&\quad \le C_3 K^{-\alpha _{1u}} \sqrt{a_1K n_c \left( r^+_{K}\right) ^{2n_c}} \le C_4 K^{\frac{1}{2}-\alpha _{1u}}\log _\rho K^c\xrightarrow [K\rightarrow \infty ]{}0, \end{aligned}$$

where $\dagger$ holds since $\alpha _{1u} > \alpha _{1\ell }$ and we used Doob’s inequality as before. This verifies (41) and, in turn, (38) for $j=2$. The proof for $j=1$ is done similarly and (37) follows. This completes the proof of (14). $\square$

## Footnotes

1 See the remark in the paragraph following (3.1) in Seneta (1969).
